# microRNA-33a-5p increases radiosensitivity by inhibiting glycolysis in melanoma

**DOI:** 10.18632/oncotarget.19014

**Published:** 2017-07-05

**Authors:** Ke Cao, Jingjing Li, Jia Chen, Li Qian, Aijun Wang, Xiang Chen, Wei Xiong, Jintian Tang, Shijie Tang, Yong Chen, Yao Chen, Yan Cheng, Jianda Zhou

**Affiliations:** ^1^ Department of Oncology of Third Xiangya Hospital, Central South University, Changsha, China; ^2^ Department of Plastic Surgery of Third Xiangya Hospital, Central South University, Changsha, China; ^3^ Department of Burn and Plastic Surgery of Second Xiangya Hospital, Central South University, Changsha, China; ^4^ Surgical Bioengineering Laboratory, Department of Surgery, UC Davis School of Medicine, Sacramento, California, USA; ^5^ Department of Dermatology of Xiangya Hospital, Central South University, Changsha, China; ^6^ Cancer Research Institute, Key Laboratory of Carcinogenesis of Ministry of Health, Central South University, Changsha, China; ^7^ Institute of Medical Physics and Engineering, Department of Engineering Physics, Tsinghua University, Beijing, China; ^8^ Department of Plastic Surgery, Second Hospital of Shantou University, Shantou, China; ^9^ Key Laboratory of Genetics and Birth Health of Hunan Province, Family Planning Institute of Hunan Province, Changsha, China; ^10^ School of Pharmaceutical sciences, Central South University, Changsha, China

**Keywords:** microRNA, radiation, glucose, HIF-1α, lactate dehydrogenase A

## Abstract

Glycolysis was reported to have a positive correlation with radioresistance. Our previous study found that the miR-33a functioned as a tumor suppressor in malignant melanoma by targeting hypoxia-inducible factor1-alpha (*HIF-1α*), a gene known to promote glycolysis. However, the role of miR-33a-5p in radiosensitivity remains to be elucidated. We found that miR-33a-5p was downregulated in melanoma tissues and cells. Cell proliferation was downregulated after overexpression of miR-33a-5p in WM451 cells, accompanied by a decreased level of glycolysis. In contrast, cell proliferation was upregulated after inhibition of miR-33a-5p in WM35 cells, accompanied by increased glycolysis. Overexpression of miR-33a-5p enhanced the sensitivity of melanoma cells to X-radiation by MTT assay, while downregulation of miR-33a-5p had the opposite effects. Finally, *in vivo* experiments with xenografts in nude mice confirmed that high expression of miR-33a-5p in tumor cells increased radiosensitivity via inhibiting glycolysis. In conclusions, miR-33a-5p promotes radiosensitivity by negatively regulating glycolysis in melanoma.

## INTRODUCTION

Malignant melanoma (MM), an aggressive skin malignancy, is the fifth most common malignant tumor in men and the seventh most common malignant tumor in women [1, 2]. Recent studies showed that the incidence of MM has increased by 3.1% per year [3]. Despite significant advances in early detection and treatment of MM, the prognosis of MM remains poor [4, 5]. Radiation is an important treatment modality for melanoma, therefore methods of increasing the radiosensitivity of melanoma cells are urgently needed.

Increased glucose uptake and accumulation of lactate are common features of cancer cells. Lactate accumulation is considered an early event of malignant tumors and promotes radiation resistance in solid tumors [6]. Sattler et al. [7] reported that tumor glycolysis is associated with inhibition of reactive oxygen species (ROS)-mediated fixation of DNA damage and induction of radioresistance. Aerobic glycolysis generates a chemically reduced milieu associated with the development of radioresistance in cancer cells, suggesting a significant therapeutic gain for combination with glycolysis inhibitors in radiotherapy [6, 8–10]. Furthermore, inhibitors of glycolysis, including 2-deoxy-d-glucose and oxamate (an inhibitor of lactate dehydrogenase A) [11, 12], obviously enhanced the sensitivity to radiotherapy. A combination of 2-deoxy-d-glucose and 6-aminonicotinamide (6-AN, an inhibitor of the pentose phosphate pathway) increased the radiosensitivity of neck cancer cells by activating ASK1/JNK/p38 MAPK signaling [13, 14]. Resistance to radiotherapy is considered to be an important factor limiting the efficacy of melanoma treatment. Therefore, developing effective targeting agents of glycolysis may provide alternative therapeutic strategies for enhancing MM radiosensitivity.

MicroRNAs (miRNAs) regulate gene expression by inhibiting gene translation or facilitating mRNA degradation [15, 16]. In recent years, accumulating evidence has revealed that miRNAs negatively modulate a variety of genes pivotal for tumor development [17–20]. miR-33a has been found to play a key role of tumor suppressor in several human cancers. For instance, Du M et al. [21] showed that miR-33a suppressed proliferation of non-small-cell lung carcinoma cells via targeting oncogene METTL3 and Kuo et al. [22] found that miR-33a functioned as a bone metastasis suppressor in lung cancer. In our previous study, we showed that miR-33a functions as a tumor suppressor by targeting HIF-1α in melanoma [23]. HIF-1α has been demonstrated to act as a key regulator in glycolysis [24]. However, the detailed functions of miR-33a-5p in MM cell radiosensitivity and the underlying molecular mechanism remains largely unclear. Not only that, related mechanisms of miRNAs negatively regulate glycolysis in melanoma radiotherapy has not been reported before. In the present study, we aimed to elucidate whether miR-33a-5p increases MM cell radiosensitivity by inhibiting glycolysis. Our data suggest that miR-33a-5p might be a promising molecular target for melanoma therapy.

## RESULTS

### Melanoma tissue and cell lines have lower miR-33a-5p expression level

**Figure 1:**
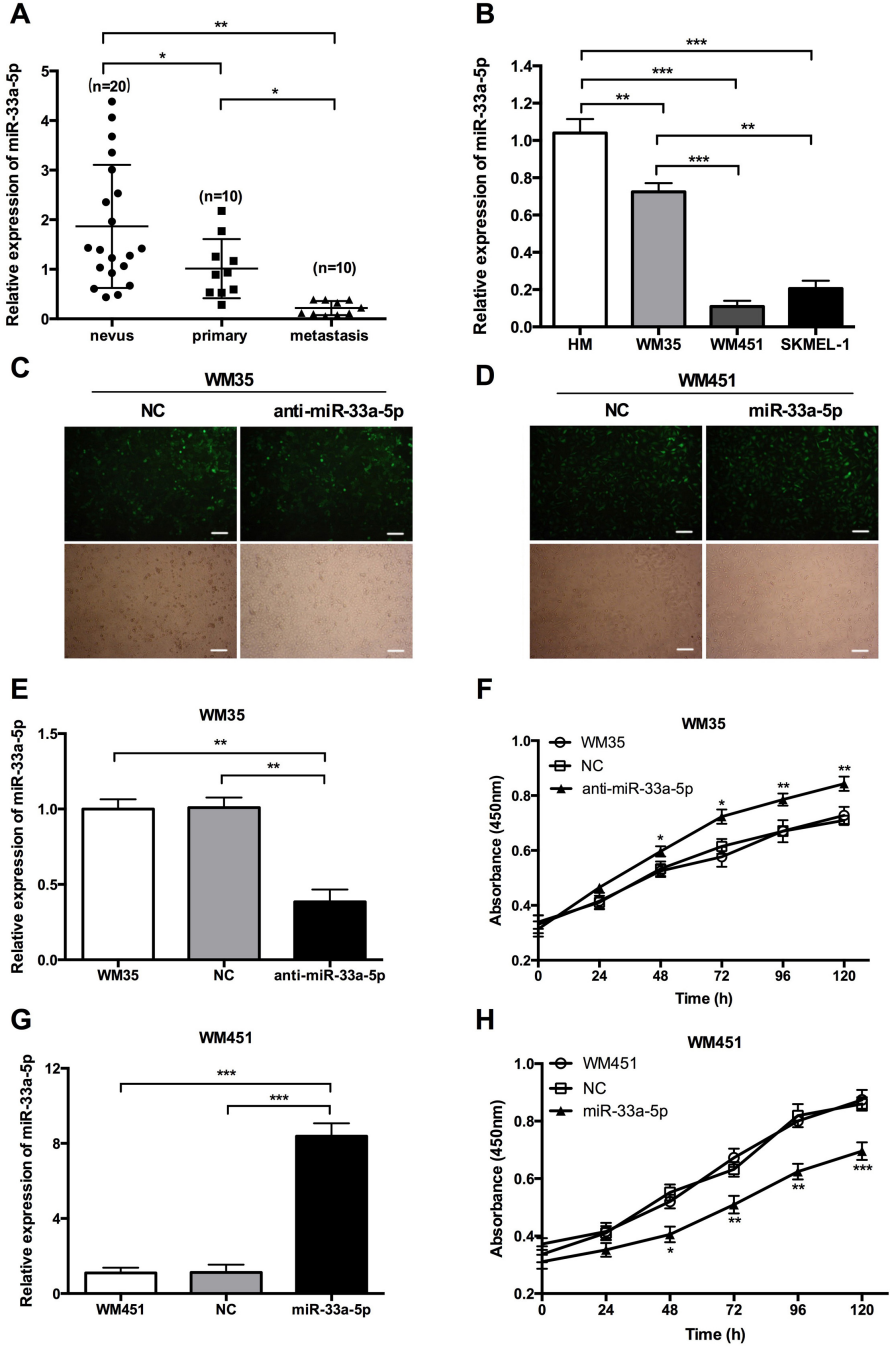
Expression of miR-33a-5p in MM tissue and cell lines (**A**) miR-33a-5p expression was significantly downregulated in MM tissue samples compared with nevus by qRT-PCR. miR-33a-5p expression was also lower in metastatic tissue compared with primary tumor tissue. (**B**) qRT-PCR was performed to analyze the expression level of miR-33a-5p in three MM cell lines (WM35, WM451, and SK-MEL-1) and human melanocytes (HM). (**C**) WM35 cells were transfected with normal control (NC) or anti-miR-33a-5p (anti-miR-33a-5p) lentivirus plasmids. Representative fluorescence microscopy (top) and bright field microscopic images (bottom) are shown in the left panel. (**D**) WM451 cells were transfected with NC (NC) or pre-miR-33a-5p (miR-33a-5p) lentivirus plasmids. Representative fluorescence microscopy (top) and bright field microscopic images (bottom) are shown in the right panel. (**E**) qRT-PCR analysis of miR-33a-5p expression relative to that in untreated WM35 cells (set as 1). (**F**) WM35 cell proliferation at the indicated time points determined by MTT assay. (**G**) qRT-PCR analysis of miR-33a-5p expression relative to that in non-treated WM451 cells (set as 1). (**H**) WM451 cell proliferation at the indicated time points determined by MTT assay. Scal bar: 50 μm. *P < 0.05, **P < 0.01, ***P < 0.001.

To explore the role of miR-33a-5p in MM, we first examined the expression level of miR-33a-5p in melanoma and nevus tissues by qRT-PCR and found that miR-33a-5p was significantly downregulated in carcinoma tissue compared with nevus tissue. Interestingly, the miR-33a-5p level in metastatic tissue specimens was much lower than that in primary tumor tissues (Figure 1A). We further examined the expression level of miR-33a-5p in three MM cell lines, WM35, WM451, and SK-MEL-1 and human melanocytes (HM). As shown in Figure 1B, the expression of miR-33a-5p was significantly downregulated in WM35, WM451 and SK-MEL-1 cells compared to HM cells. Based on these preliminary data, we chose WM35 and WM451 cells to further investigate the role of miR-33a-5p in MM *in vitro*.

### miR-33a-5p inhibits the viability of melanoma cells

The expression of miR-33a-5p was low in WM35 and WM451 cells, but much lower in WM451 cells, we transfected WM451 cells with pre-miR-33a-5p labeled green fluorescent protein plasmid and WM35 cells with anti-miR-33a-5p labeled green fluorescent protein plasmid. The transfection efficiency was evaluated by fluorescence microscopy and qRT-PCR. The green fluorescence representes the transfection efficiency of each plasmid. Over 80% of WM35 (Figure 1C) and WM451 (Figure 1D) cells were stained in green fluorescence, which suggesting the transfection efficiency was satisfied. The results of qRT-PCR show that miR-33a-5p expression in WM35 cells was notably reduced after transfection with anti-miR-33a-5p plasmid (Figure 1E) whereas expression in WM451 cells was increased after transfection with pre-miR-33a-5p plasmid (Figure 1G), confirming that the transfections were successful.

Cell viability in each group was determined by MTT assay and found that cell proliferation was upregulated after inhibition of miR-33a-5p in WM35 cells (Figure 1F) but was downregulated after overexpression of miR-33a-5p in WM451 cells (Figure 1H), suggesting that miR-33a-5p plays an inhibitory role in MM cell proliferation.

### miR-33a-5p inhibits glycolysis in melanoma cells

**Figure 2:**
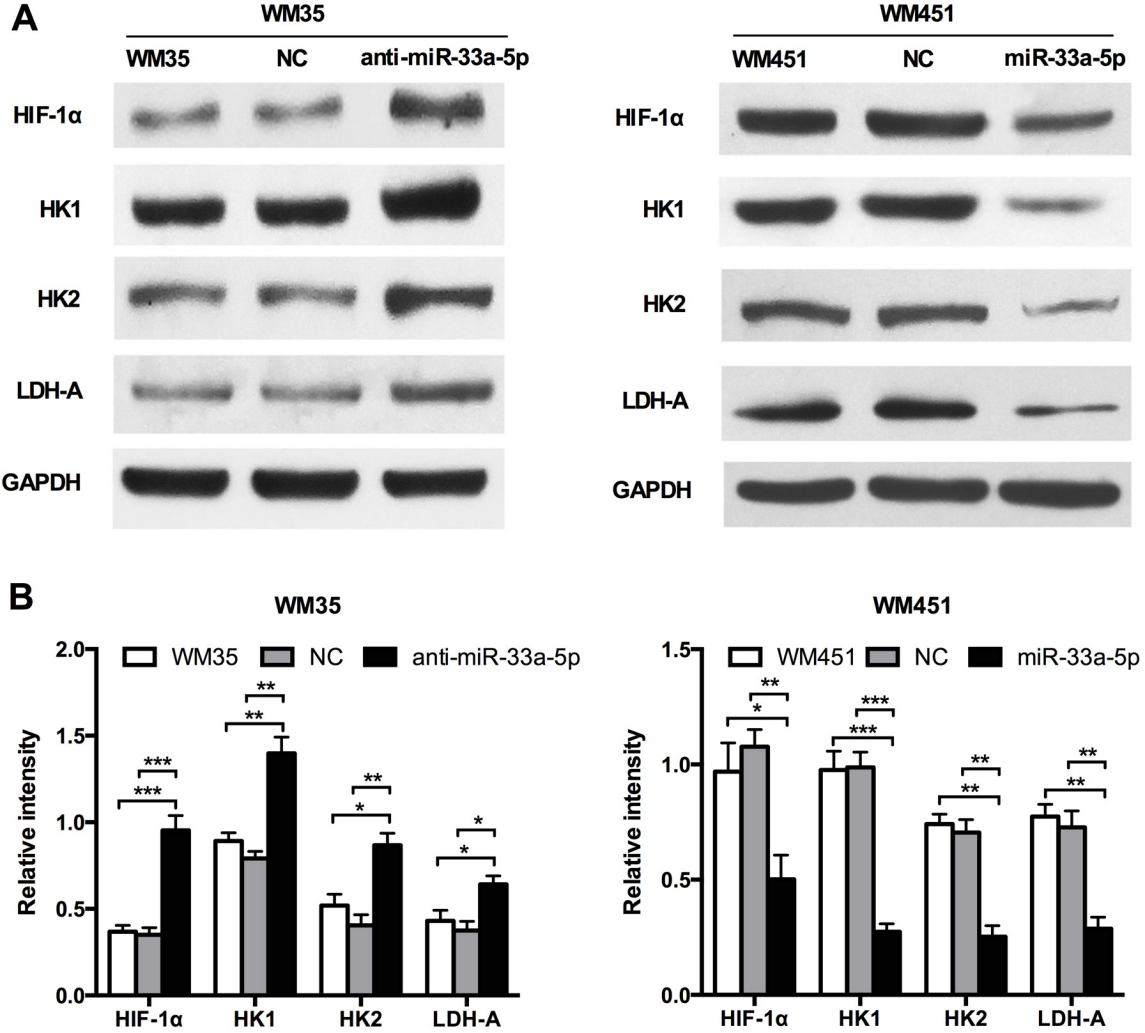
miR-33a-5p targets the glycolysis signaling pathway in MM (**A**) Western blot analysis of HIF-1α, HK1, HK2, and LDH-A protein expression in WM35 and WM451 cells. GAPDH protein expression was used as a control for input and normalization. (**B**) Quantitative graph showing the image intensity for WM35 and WM451 cells shown in A. *P < 0.05, **P < 0.01, ***P < 0.001.

As glycolysis functions as a promoter in radiosensitivity, we determined the glycolysis level in each group of cells. HIF-1 is a key regulator of cellular and systemic homeostatic responses to hypoxia through the activation of gene transcription, and is involved in energy metabolism, angiogenesis, and apoptosis [25, 26]. In our previous study, we confirmed that HIF-1α is a direct gene target of miR-33a in melanoma [22]. Furthermore, HIF-1α has been demonstrated to act as a key regulator in glycolysis, HIF-1α is the best-known transcriptional regulators controlling expression of glycolysis genes, such as HK1, HK2, and LDHA, whose expression levels are highly elevated in cancer cells [27]. As shown in Figure 2A, 2B, the expression of HIF-1α, HK1, HK2, and LDH-A was notably increased after inhibition of miR-33a-5p in WM35 cells, but reduced after miR-33a-5p overexpression in WM451 cells. These results suggest that miR-33a-5p inhibits glycolysis in MM cells, and that HIF-1α is involved in this inhibitory activity.

**Figure 3:**
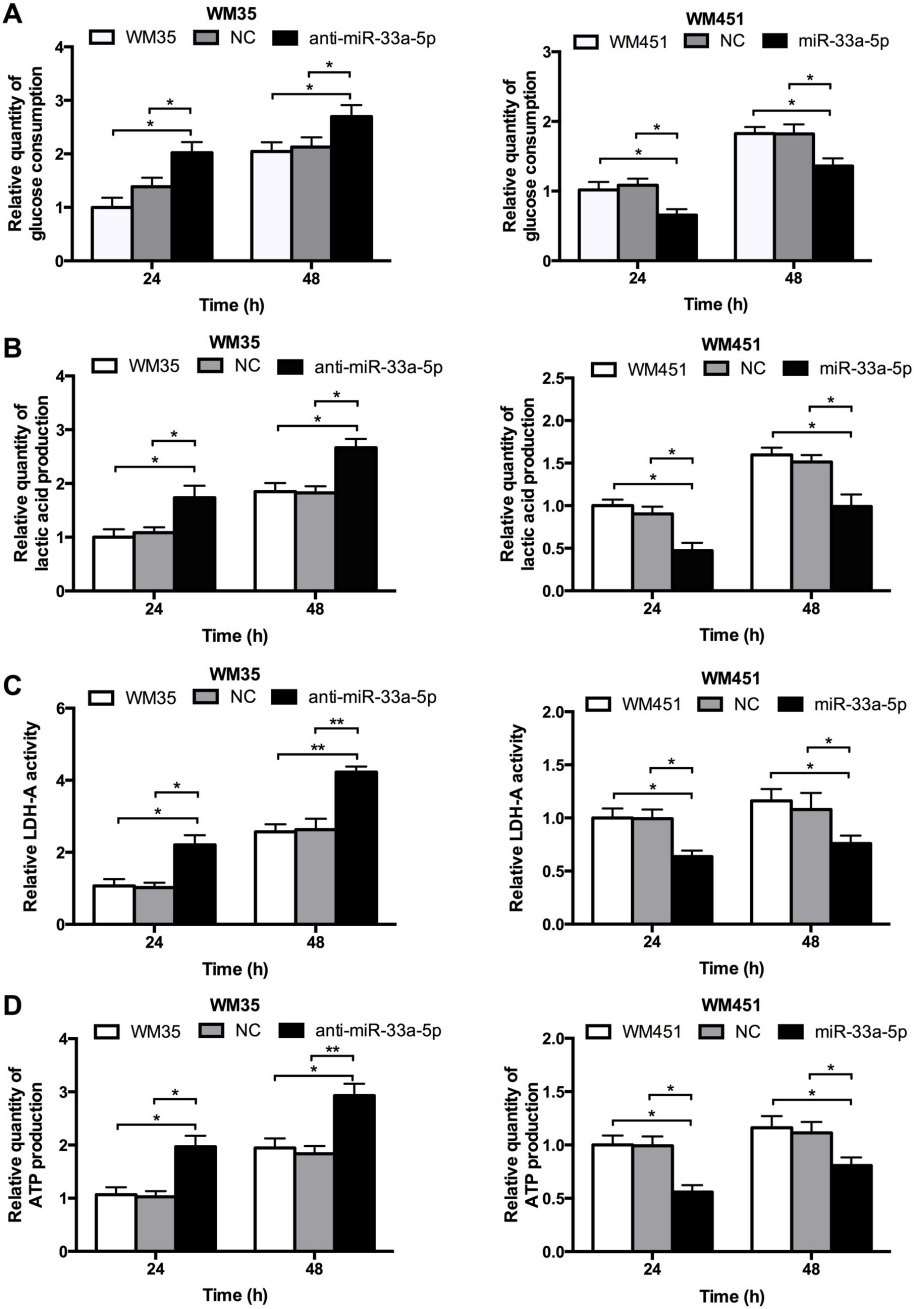
miR-33a-5p plays an inhibitory role in glycolysis in MM cells (**A**) Glucose consumption was measured by microplate assay, with the value for non-treated cells set as 1. (**B**) Lactic acid production in WM35 and WM451 cells. Non-treated cells were used for normalization. (**C**) Activity of LDH-A was analyzed by activity gel assay. (**D**) ATP production was measured using a luciferin/luciferase assay.*P < 0.05, **P < 0.01.

Glucose uptake and LDH-A activity are main indicators of glucose metabolism. Lactic acid production, and ATP production were also measured to evaluate the glycolysis level, which are main products of glycolysis. As shown in Figure 3A–3D, lactic acid production, LDH-A activity, and ATP production were notably upregulated after inhibition of miR-33a-5p in WM35 cells, but all four parameters were downregulated after miR-33a-5p overexpression in WM451 cells. These findings further suggested that miR-33a-5p plays a suppressive role in the regulation of glycolysis in MM cells.

### HIF-1α is involved in miR-33a-5p-mediated glycolysis in melanoma cells

**Figure 4:**
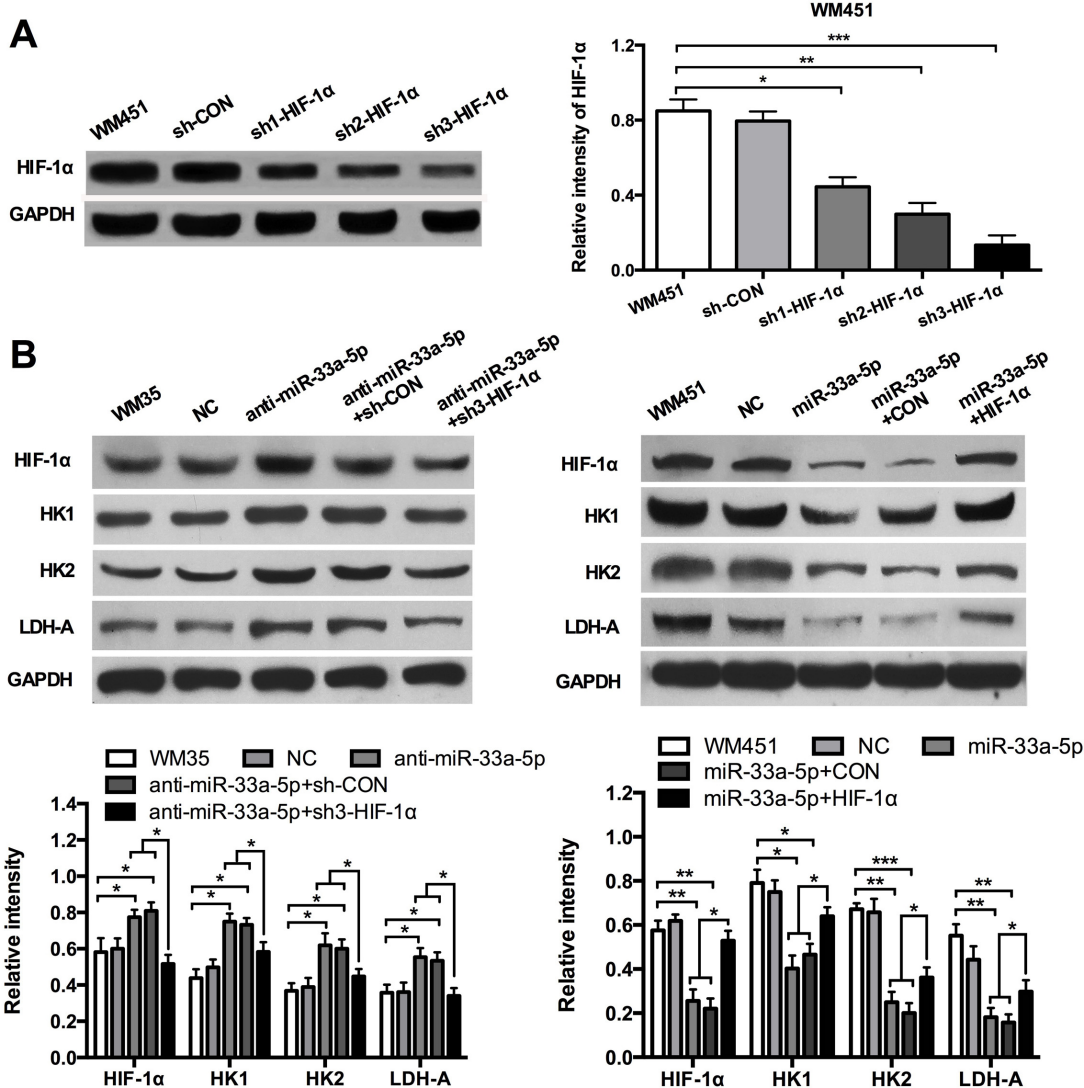
HIF-1α restores the inhibitory effect of miR-33a-5p on glycolysis (**A**) Three shRNA expression plasmids targeting HIF-1α were transfected into WM451 cells, respectively. Western blotting results revealed that the third shRNA sequence had the best inhibitory effect. (**B**) WM35 cells were transfected with anti-miR-33a-5p plasmid, or co-transfected with anti-miR-33a-5p and sh-HIF-1α plasmids. WM451 cells were transfected with pre-miR-33a-5p plasmid, or co-transfected with pre-miR-33a-5p and HIF-1α plasmids. HIF-1α, HK1, HK2, and LDH-A protein expression was measured by western blotting. GAPDH protein expression was used as a control for input and normalization. *P < 0.05, **P < 0.01, ***P < 0.001.

To further investigate whether HIF-1α acts as a downstream effecter in miR-33a-5p-mediated glycolysis in MM cells, we constructed 3 shRNA (small hairpin RNA) expression plasmids targeting HIF-1α, and then transfected into WM451 cells by lipofectamine methods. Western blotting was performed to detect the expression of HIF-1α protein (Figure 4A). Western blotting results revealed that the third shRNA sequence had the best inhibitory effect, sh3-HIF-1α plasmid was chose for further investigation. The cell proliferation after co-transfection with anti-miR-33a-5p and shHIF-1α plasmids or miR-33a-5p and HIF-1α plasmids were presented in supplementary materials. The results showed that HIF-1α significantly restored the inhibitory effect of miR-33a-5p (Supplementary Figure 1). WM35 cells were transfected with anti-miR-33a-5p plasmid or co-transfected them with two lasmids expressing anti-miR-33a-5p and sh3-HIF-1α, respectively. WM451 cells were transfected with pre-miR-33a-5p plasmid, or co-transfected them with two plasmids expressing pre-miR-33a-5p and HIF-1α, respectively. Glycolysis level was examined in each group. As shown in Figure 4B, downregulation of HIF-1α reversed the promoting effect of miR-33a-5p inhibition on glycolysis in WM35 cells, whereas upregulation of HIF-1α reversed the inhibitory effect of miR-33a-5p overexpression on glycolysis in WM451 cells.

**Figure 5:**
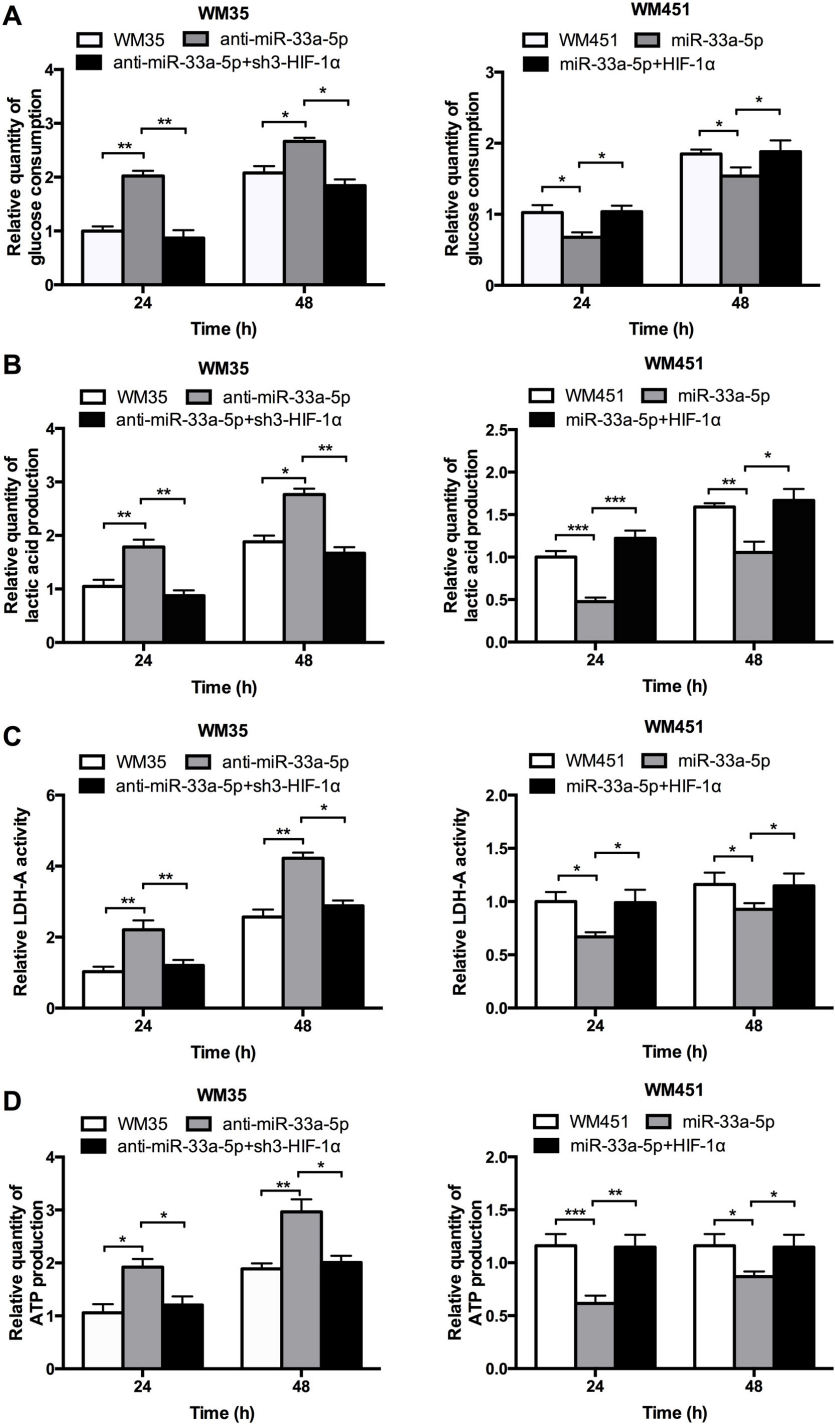
miR-33a-5p/HIF-1α axis plays an important role on glycolysis (**A**) Glucose consumption was measured by microplate assay, with the value for non-treated cells set as 1. (**B**) Lactic acid production was detected in WM35 and WM451 cells. Untreated cells were used for normalization. (**C**) Activity of LDH-A was analyzed by activity gel assay. (**D**) ATP production was measured using a luciferin/luciferaseassay.*P < 0.05, **P < 0.01, ***P < 0.001.

Glucose consumption, lactic acid production, LDH-A activity, and ATP production were measured to evaluate the glycolysis level. Figure 5A–5D shows that the glucose consumption, lactic acid production, LDH-A activity, and ATP production were notably reduced in WM35 cells after co-transfection with anti-miR-33a-5p and sh3-HIF-1α plasmids but were significantly upregulated in WM451cells after co-transfection with pre-miR-33a-5p and HIF-1α plasmids. These findingsconfirmed that miR-33a-5p plays an inhibitory role in glycolysis in MM cells by negatively regulating HIF-1α.

### miR-33a-5p enhances radiosensitivity of melanoma cells to X-radiation

**Figure 6:**
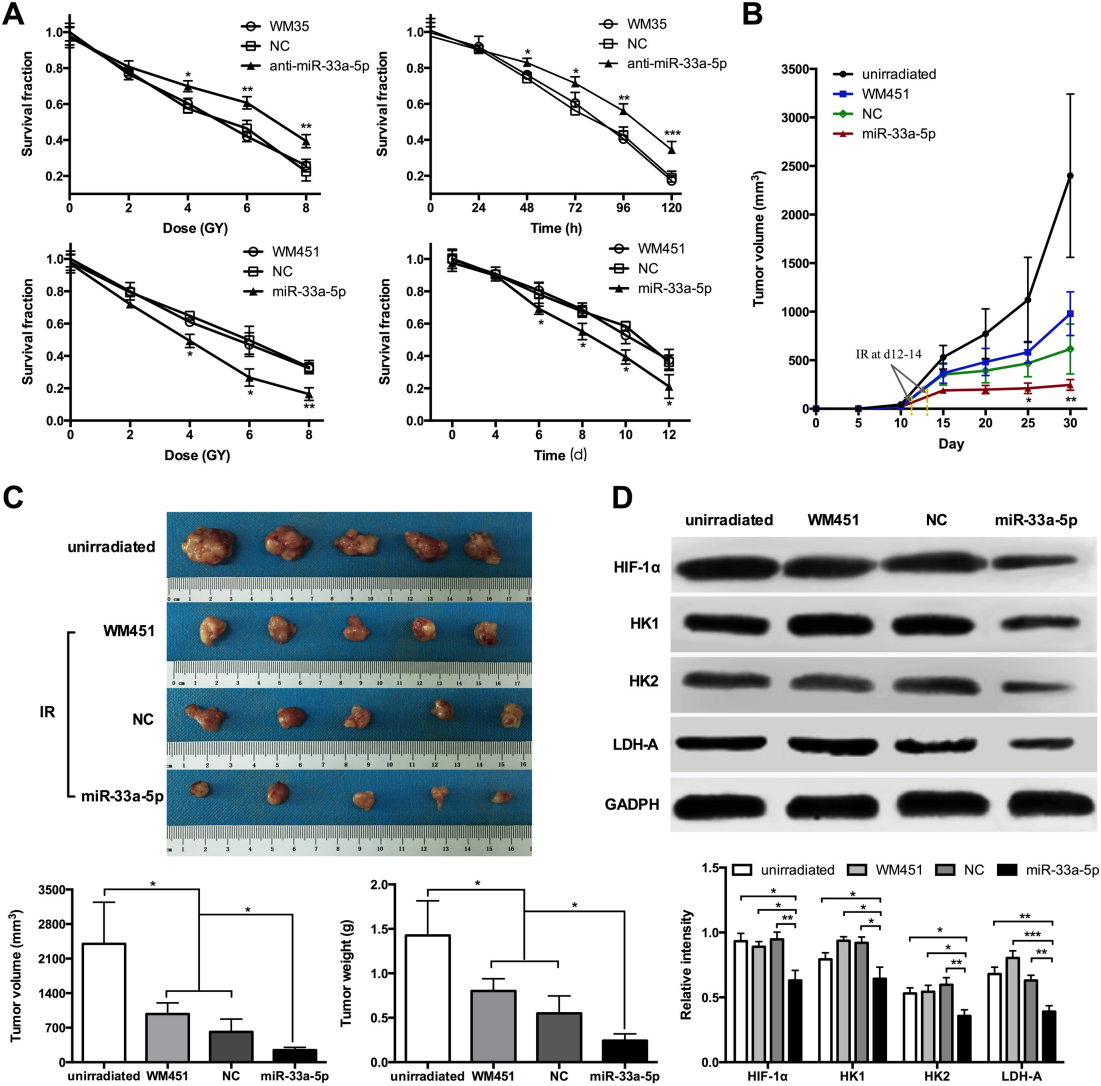
miR-33a-5p increases cell radiosensitivity by negatively regulating glycolysis in MM (**A**) MTT assay was performed to measure the cell survival rate of WM35 and WM451 cells at 8 days under 0, 2, 4, 6, and 8 Gy irradiation. The survival rate was significantly lower in the miR-33a-5p group compared with the non-transfected and control groups. MTT assay was also performed to observe the cell survival rate at the indicated time points under 4 Gy X-ray irradiation. (**B**) Growth curve of nude mice with different levels of miR-33a-5p expression. Day 0 means the day of injection of tumor cells. Arrows indicated the day radiotherapy began for mice in WM451, NC, and miR-33a-5p groups. (**C**) Representative images of subcutaneous tumors at day 30. Tumor volume and weight of WM451 xenografts in the unirradiated, WM451, NC, and miR-33a-5p groups. The results were analyzed using one-way ANOVA. (**D**) Western blotting of HIF-1α, HK1, HK2, and LDH-A protein expression in WM451 xenografts. GAPDH protein expression was used as a control for input and normalization. IR: irradiation. *P < 0.05, **P < 0.01, ***P < 0.001.

As indicated in Figure 6A, administration of increasing doses of radiation resulted in a dose-dependent downward trend in the cell survival rate of WM35 and WM451. In particular, the survival rate of WM35 cells was much higher in anti-miR-33a-5p group, while WM451 cells was lower in the miR-33a-5p group (*P* < 0.05). Considering the cell survival rate under 4 Gy X-ray irradiation were ranged 50% to 60% in both untreated WM35 and WM451 cells, which could act as an ideal observation window for further exploration. The MTT assay was performed to observe the cell survival rate at the indicated time points under 4-Gy X-ray irradiation. The results revealed a time-dependent downward trend in the cell survival rate. The survival rate of WM35 cells was higher after downregulation of miR-33a-5p, while WM451 cells was lower after overexpression of miR-33a-5p (*P* < 0.05). These experimental data indicated that overexpression of miR-33a-5p might promote radiosensitivity of MM cells.

### miR-33a-5p increases cell radiosensitivity by negatively regulating glycolysis *in vivo*

Nude mice were subcutaneously inoculated with WM451 cells, WM451 cells transfected with blank empty vector (NC), or WM451 cells transfected with pre-miR-33a-5p (miR-33a-5p) vector. The results showed that it took 12–14 days after tumor implantation for the tumor volume of each group reached 150 mm^3^. After tumor development, the mice were treated with irradiation at a total dose of 10 Gy. The growth curve of each group was showed at Figure 6B, which revealed that the tumor volume of nude mice in the miR-33a-5p group was significantly lower than that in unirradiated, WM451, and NC groups (*P* < 0.05). The average volume of the tumors (Figure 6C) in the unirradiated, WM451, and NC groups was 2400.83 ± 841.23, 979.15 ± 225.26 and 615.96 ± 257.78 mm^3^, respectively, which was notably higher than that in the miR-33a-5p group (246.07 ± 55.72 mm^3^). The average tumor mass (Figure 6C) in the unirradiated, WM451, and NC groups was 1.43 ± 0.39, 0.80 ± 0.14 and 0.55 ± 0.20 g, respectively, which was also higher than that in the miR-33a-5p group (0.24 ± 0.08 g). To analyze the relationship between radiosensitivity and glycolysis, western blot analysis was performed to measure the HIF-1α, HK1, HK2, and LDH-A protein expression levels in the xenograft tumor (Figure 6D). HIF-1α, HK1, HK2, and LDH-A proteins were significantly downregulated after overexpression of miR-33a-5p. Therefore, melanoma cells with high miR-33a-5p expression level increased radiosensitivity accompanied by downregulation of glycolysis *in vivo*.

## DISCUSSION

Increasing evidence indicates that microRNAs are associated with tumor radiosensitivity. Zhu et al. [28] found that downregulation of miR-21 enhanced radiosensitivity in nasopharyngeal carcinoma. Another study confirmed that miR-622 induced radioresistance by targeting Rb in colorectal cancer cells [29]. Liu et al. [30] reported that miR-18a increased radiosensitivity by promoting radiation-induced apoptosis in cervical cancer cells. In our previous study, we demonstrated that miR-33a functions as a tumor suppressor by targeting HIF-1α in melanoma cells [23]. The present study aim to investigate whether miR-33a-5p restores malanoma radioresistance.

We found that miR-33a-5p was significantly downregulated in melanoma tissue compared with nevus tissue. Interestingly, miR-33a-5p expression was much lower in metastatic tissue than in primary tissues. miR-33a-5p expression was also notably reduced in MM cells. These results verified those reported previously by us. To further explore the inhibitory effect of miR-33a-5p *in vitro*, WM35 and WM451 cells were selected for subsequent experiments. MTT assays revealed that cell proliferation was upregulated after inhibition of miR-33a-5p in WM35 cells, but notably downregulated after overexpression of miR-33a-5p in WM451 cells. Therefore, miR-33a-5p suppresses proliferation in MM cells.

Although less efficient than oxidative phosphorylation in the yield of adenosine triphosphate, glycolysis can more rapidly provide cancer cells with energy and the metabolic intermediates needed for macromolecular biosynthesis, conferring advantages to cancer cells under conditions of limited nutrient supply [31]. Therefore, targeting glycolysis seems to be an effective tumor therapy strategy. After showing that miR-33a-5p could inhibit MM cell proliferation, we focused on the effect of miR-33a-5p on glycolysis in MM cells. The three key enzymes of glycolysis are HK1, HK2, and LDAH [32]. We found that the expression of HIF-1α, HK1, HK2, and LDH-A was increased after inhibition of miR-33a-5p in WM35 cells, but reduced after miR-33a-5p overexpression in WM451 cells. Moreover, knockdown of miR-33a-5p promoted glycolysis, as measured by glucose consumption, lactic acid production, LDH-A activity, and ATP production, whereas upregulation of miR-33a-5p inhibited glycolysis in MM cells. In our previous study, we confirmed that miR-33a-5p functions as tumor suppressor mainly via directly binding HIF-1α 3’UTR [23]. HIF-1α is one of hypoxic-related genes helps tumor cells adapting to the low nutrient and oxygen microenviroment. There is an obvious positive correlation between HIF-1α and glycolysis during tumorigenesis [33, 34]. miR-33a-5p plays an inhibitory role in glycolysis in melanoma, suggesting HIF-1α may involve in this activity. We further confirmed that upregulation of HIF-1α reversed the inhibitory effect of miR-33a-5p overexpression on glycolysis in WM451 cells, whereas downregulation of HIF-1α reversed the promoting effect of miR-33a-5p inhibition on glycolysis in WM35 cells. HK1/HK2 as well as LDAH was proved upregulated by HIF-1α overexpression, while was inhibited by knock-down HIF-1α, which were basically in accord with previous studies [35]. HK2 is considered as a downstream of HIF-1α in fermentation, which acts as a rate-limiting enzyme and involves in one irreversible step of glycolysis [36]. The results mentioned above suggesting that miR-33a-5p represses glycolysis mainly via inhibition of HIF-1α in melanoma.

**Figure 7:**
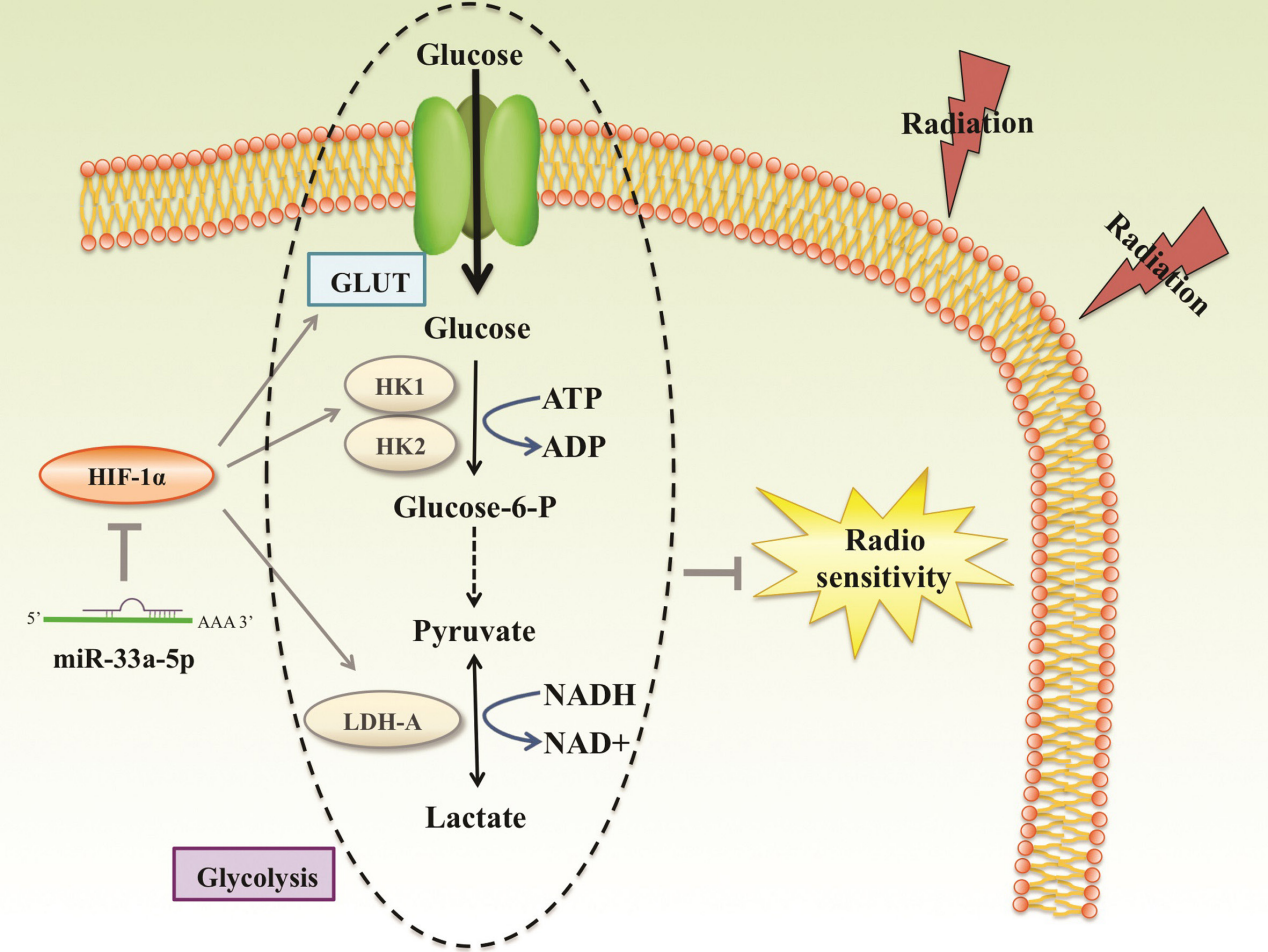
Reported and potential mechanisms for the regulation of the radiosensetivity by miR-33a-5p in MM miR-33a-5p inhibits the process of glycolysis mainly via negatively regulating HIF-1α, a closely related gene of glycolysis. Glycolysis products such as pyruvate and lactate played important roles in radioresistance. miR-33a-5p increased MM cell radiosensetivity account for down-regulation glycolysis level.

Radioresistance is one of major problems encountered in cancer therapy. Previous studies have strongly suggested that glycolysis leads to radiation resistance of cancer cells [37]. HIF-1α has recently been shown being participated in tumor glycometabolism. Jiang S et al. [38] found that HIF-1α was upregulated by miR-21 in radioresistant non-small cell lung cancer cells. Considering miR-33a-5p acts as an upstream factor of HIF-1α, MTT assays were performed to measure the cell survival rate of MM cells under X-ray irradiation. The results indicated that the radiosensitivity of MM cells was enhanced by overexpression of miR-33a-5p. Accordingly, we suggest that the radiosensitizing effects of miR-33a-5p in MM cells *in vitro* may be mediated by suppression of glycolysis. WM35 cells are lowly tumorigenic and even non-tumorigenic in nude mice [39], Therefore only WM451 cells were performed to explorethe antitumor effect of miR-33a-5p. Finally, *in vivo* xenograft experiments revealed that the tumor burden of melanoma was reduced by radiotherapy, and high expression of miR-33a-5p in MM cells increased radiosensitization accompanied by downregulation of glycolysis. These findings indicating that miR-33a-5p functions as a tumor suppressor by inhibiting HIF-1α-mediated glycolysis (Figure 7). However, there are some questions remain to be clarify. Understanding why these cells under-expressed miR-33a-5p is the next step in our further education.

In summary, we suggest that miR-33a-5p promotes radiosensitivity by negatively regulating glycolysis in MM cells. Therefore, targeting the miR-33a-5p/glycolysis pathway may provide a new direction for radiotherapy of melanoma.

## MATERIALS AND METHODS

### Reagents

Glucose-Dulbecco's modified Eagle's medium (DMEM), fetal bovine serum (FBS), Trizol reagent, miRNA reverse transcription kit, and Lipofectamine 2000 were purchased from Life Technologies (Waltham, MA). BCA protein assay kits were purchased from Beyotime (Haimen, China). Pre-miR-33a-5p lentivirus plasmid, anti-miR-5p lentivirus plasmid, HIF-1α overexpressing plasmid, and HIF-1α shRNA plasmid were purchased from DingGuoChangShengBiotech (Beijing, China). Sequences of the insert in plasmids were listed in Supplementary Table 1. miRNA qRT-PCR Detection Kit was purchased from GeneCopoeia, Rockville, MD, USA. RevertAid^™^ H Minus First Strand cDNA Synthesis Kit was purchased from Fermentas. MTT was purchased from Biosharp (Hefei, Anhui, China). The Power SYBR Green kit was purchased from TOYOBO (Osaka, Japan). The glucose assay kit, lactate assay kit, and LDH activity assay kit were from Biovision (Milpitas, CA). The ATP assay Kit was from Beyotime Biotechnology (Shanghai). Mouse anti-HIF-1α monoclonal antibody, mouse anti-glyceraldehyde-3-phosphate dehydrogenase (GAPDH) monoclonal antibody, and rabbit anti-mouse secondary antibody were purchased from Abcam (Cambridge, UK) and anti-lactate dehydrogenase A (LDH-A) antibody was from Epitomics (Burlingame, CA).

### Samples

Twenty melanoma and match nevus tissues were collected by the Department of Pathology at the Second Xiangya Hospital and Third Xiangya Hospital, Central South University between 2004 and 2014, following informed consent from each of the patients and approval by the Ethics Committee of Third Xiangya Hospital, Central South University. None of the melanoma patients received chemotherapy or radiotherapy before surgery. All specimens were fixed in 10% neutral formalin and embedded in paraffin.

### Cell culture

Human malignant melanoma cell lines A375, WM35, WM451, and SK-MEL-1 were obtained from the Cell Bank of Central South University, Changsha, China. Primary human melanocytes (HM) were purchased from Shanghai XinYu Biological Technology Co., Ltd (Shanghai, China). Cells were cultured in DMEM supplemented with 10% FBS at 37°C in a humidified incubator containing 5% CO_2_.

### Transfection

Transfection was performed using Lipofectamine 2000 in accordance with the manufacturer's instructions. In brief, for functional analysis MM cells were transfected with the pre-miR-33a-5p lentivirus plasmid, anti-miR-33a-5p lentivirus plasmid, HIF-1α overex pressing plasmid, or HIF-1α shRNA plasmid.

### Real-time RT-PCR assay

Total RNA was extracted with Trizol agent in accordance with the manufacturer's instructions. For the analysis of miRNA expression, a miRNA reverse transcription kit was used to convert RNA into cDNA according to the manufacturer's protocol. Real-time PCR was performed using a miRNA qRT-PCR Detection Kit on an ABI 7500 thermocycler (Life Technologies). The *U6* gene was used as an endogenous control. For the analysis of mRNA expression, Revert Aid™ H Minus First Strand cDNA Synthesis Kit was used to convert RNA into cDNA and real-time PCR was performed using a Power SYBR Green kit on an ABI 7500 thermocycler. *GAPDH* was used as an endogenous control. For both mRNA and miRNA, the relative expression was analyzed by the 2^−ΔΔCt^ method. The primers used were as follows: miR-33a-5p, 5′-GATCCTCAGTGCATTGTAGTTGC-3′ and reverse 5′-CTCTGTCTCTCGTCTTGTTGGTAT-3′; U6, 5′-ATTGGAACGATACAGAGAAGATT-3′ and reverse 5′-GGAACGCTTCACGAATTTG-3′.

### Cell viability assay

For all groups, 10,000 cells per well were plated in a 96-well plate. After treatment, the plates were incubated for 0, 24, 48, 72, 96 or 120 h at 37°C, 5% CO_2_. To assess cell viability, the MTT assay was performed according to the manufacturer's manual. Briefly, 10 μl of 5 mg/ml MTT reagent in PBS was added to each well and incubated for 4 h at 37°C, 5% CO_2_. The supernatant was removed and 100 μl of DMSO was added. Absorbance was detected at 450 nm with a Microplate Reader (Bio-Rad, USA). Each assay was performed in triplicate wells and repeated three times.

### Western blotting

Protein was extracted from the indicated cells using RIPA lysis buffer. Protein assay reagents (Beyotime) were used to measure the protein concentration. A total of 60 μg of protein was separated with 12% SDS-PAGE and transferred to a PVDF membrane, which was blocked in 5% nonfat dried milk in PBS at room temperature for 2 h. The membrane was incubated with mouse anti-HIF-1α antibody or mouse anti-GAPDH monoclonal antibody overnight at 4°C and then with goat anti-mouse secondary antibody for 2 h. Enhanced chemiluminescence reagent was used to detect the signal on the membrane. Data were analyzed by densitometry using Image-Pro plus software 6.0 and normalized to GAPDH expression. The western blot experiments were repeated at least three times.

### Measurement of glycolysis

Glucose consumption was detected using a glucose assay kit according to the manufacturer's protocol. The absorbance at 570 nm was measured with a plate reader (Thermo Multiskan MK3 spectrophotometer; Thermo Fisher Scientific, Waltham, MA). Lactate levels in the culture media were determined using a lactate assay kit according to the manufacturer's protocol.

Total intracellular LDH activity was examined using an LDH activity assay kit according to the manufacturer's instructions. The results were normalized to the total protein concentration. Briefly, 2 × 10^5^ cells per well were plated in a 24-well plate for 48 h. The cells were collected, washed, and protein was extracted for measurement of LDH-A activity.

Cellular ATP was measured using a luciferin/luciferase assay kit. Cells were permeabilized prior to the addition of luciferin substrate and luciferase. Bioluminescence was assessed on an LUMATLB9507 luminometer (EG&G Berthold, Bad Wildbad, Germany) and cellular ATP content (nmol/mg) was determined using a curve for the luminescence of standard dilutions of ATP. ATP content was normalized to protein content.

### Radiation studies

A Varian Truebeam linear accelerator (Varian, USA) was used for X-ray irradiation. Cells at a density of 3 × 10^3^ cells/well were irradiated with different doses of X-rays (100 cm, 4 Gy/min) after 48 h of transfection. Animal experimentation was performed according to NIH Animal Care guidelines, and the entire experiment was approved by the Ethics Committee of Third Xiangya Hospital, Central South University. Male Balb/c Nu/nu mice aged approximately 4–6 weeks were obtained from Third Xiangya Hospital of Central South University and 2 × 10^6^ WM451 cells were subcutaneously injected into the flanks of the mice. When the tumor volume reached 150 mm^3^, the mice were treated with irradiation at a total dose of 10 Gy for 5 consecutive days. Nude mice were sacrificed 30 days after tumor implantation, and tumor volume and weight were measured. Tumor volume was calculated using the formula V (cm^3^) = 0.5 × a × b^2^, where a is the maximum long diameter and b is the maximum transverse diameter.

### Statistical analysis

All experiments were repeated at least three times, and the results are expressed as the mean ± SD (*n* = 3). SPSS18.0 software (SPSS, Inc., Chicago, IL, USA) was used to perform statistical analysis. Statistical analysis of differences was performed by one-way analysis of variance (ANOVA). *P < 0.05 was considered statistically significant.

## SUPPLEMENTARY MATERIALS FIGURES AND TABLES




