# Supplementary material for: microRNA-33a-5p increases radiosensitivity by inhibiting glycolysis in melanoma
Source: Oncotarget. 2017 Jul 5;8(48):83660–72. doi: 10.18632/oncotarget.19014 (PMC5663544; doi:10.18632/oncotarget.19014)
Supplement: Supplementary file 1 [file oncotarget-08-83660-s001.pdf]

microRNA-33a-5p increases radiosensitivity by inhibiting glycolysis in melanoma

Supplementary Materials

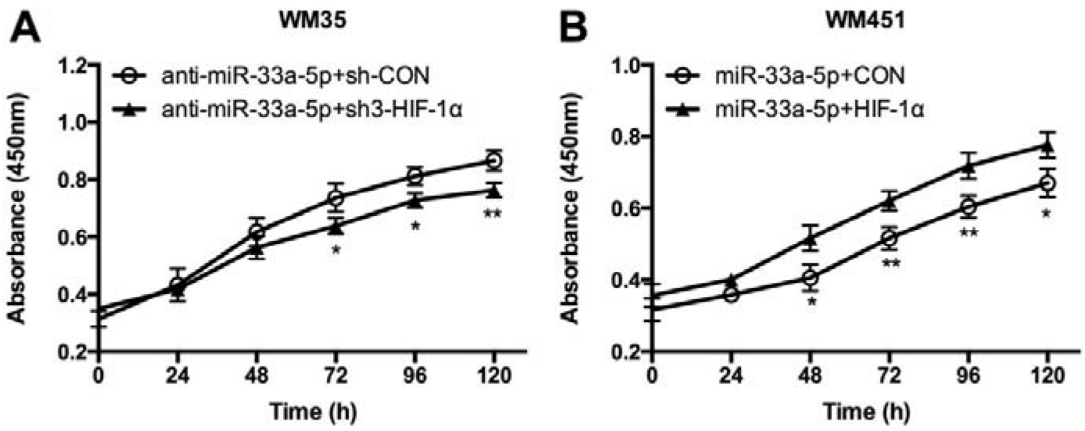

Supplementary Figure 1: To validate the function of miR-33a-5p/HIF-1α axis in cell proliferation, MTT assay was performed to measure cell viability at the indicated time points. (**A**) WM35 cells were co-transfected with anti-miR-33a-5p lentivirus plasmids and an empty shRNA plasmids or co-transfected with anti-miR-33a-5p lentivirus plasmids and sh3-HIF-1α plasmids, respectively. MTT assay results revealed that down-regulation of HIF-1α remarkably reversed the pro-proliferation by inhibiting miR-33a-5p. (**B**) The proliferation was significantly promoted after co-transfection with miR-33a-5p lentivirus plasmids and HIF-1α plasmids, suggesting overexpression HIF-1α restored the viability of WM451 cells.

Supplementary Table 1: Sequences of the insert in plasmids. See Supplementary_Table_1
