# Supplementary material for: microRNA-33a-5p increases radiosensitivity by inhibiting glycolysis in melanoma
Source: Oncotarget. 2017 Jul 5;8(48):83660–72. doi: 10.18632/oncotarget.19014 (PMC5663544; doi:10.18632/oncotarget.19014)
Supplement: Supplementary file 2 [file oncotarget-08-83660-s002.docx]

Supplementary Table 1. Sequences of the insert in plasmids

| Plasmid | The sequence |
| --- | --- |
| Pre-miR-33a-5p | F: 5’-GATCC**GTGCATTGTAGTTGCATTGCA**TTCAAGAGATGCAATGCAACTACAATGCACTT TTTTG-3’ |
|  | R: 5’-AATTCAAAAAAGTGCATTGTAGTTGCATTGCATCTCTTGAATGCAATGCAACTACAATG CACG-3’ |
| anti-miR-5p | F: 5’-GATCC**TGCAATGCAACTACAATGCAC**TTCAAGAGAGTGCATTGTAGTTGCATTGCATTT TTTG－3’ |
|  | R: 5’-AATTCAAAAAATGCAATGCAACTACAATGCACTCTCTTGAAGTGCATTGTAGTTGCATTGC ACACG－3’ |
| NC | F: 5’-GATCCG**TTCTCCGAACGTGTCACGT**TTCAAGAGAACGTGACACGTTCGGAGAACTTTT TTG-3’ |
|  | R: 5’-AATTCAAAAAAGTTCTCCGAACGTGTCACGTTCTCTTGAAACGTGACACGTTCGGAGAA CG -3’ |
| HIF-1α (2481bp) | ATGGAGGGCGCCGGCGGCGCGAACGACAAGAAAAAGATAAGTTCTGAACGTCGAAAAGAAAAGTCTCGAGATGCAGCCAGATCTCGGCGAAGTAAAGAATCTGAAGTTTTTTATGAGCTTGCTCATCAGTTGCCACTTCCACATAATGTGAGTTCGCATCTTGATAAGGCCTCTGTGATGAGGCTTACCATCAGCTATTTGCGTGTGAGGAAACTTCTGGATGCTGGTGATTTGGATATTGAAGATGACATGAAAGCACAGATGAATTGCTTTTATTTGAAAGCCTTGGATGGTTTTGTTATGGTTCTCACAGATGATGGTGACATGATTTACATTTCTGATAATGTGAACAAATACATGGGATTAACTCAGTTTGAACTAACTGGACACAGTGTGTTTGATTTTACTCATCCATGTGACCATGAGGAAATGAGAGAAATGCTTACACACAGAAATGGCCTTGTGAAAAAGGGTAAAGAACAAAACACACAGCGAAGCTTTTTTCTCAGAATGAAGTGTACCCTAACTAGCCGAGGAAGAACTATGAACATAAAGTCTGCAACATGGAAGGTATTGCACTGCACAGGCCACATTCACGTATATGATACCAACAGTAACCAACCTCAGTGTGGGTATAAGAAACCACCTATGACCTGCTTGGTGCTGATTTGTGAACCCATTCCTCACCCATCAAATATTGAAATTCCTTTAGATAGCAAGACTTTCCTCAGTCGACACAGCCTGGATATGAAATTTTCTTATTGTGATGAAAGAATTACCGAATTGATGGGATATGAGCCAGAAGAACTTTTAGGCCGCTCAATTTATGAATATTATCATGCTTTGGACTCTGATCATCTGACCAAAACTCATCATGATATGTTTACTAAAGGACAAGTCACCACAGGACAGTACAGGATGCTTGCCAAAAGAGGTGGATATGTCTGGGTTGAAACTCAAGCAACTGTCATATATAACACCAAGAATTCTCAACCACAGTGCATTGTATGTGTGAATTACGTTGTGAGTGGTATTATTCAGCACGACTTGATTTTCTCCCTTCAACAAACAGAATGTGTCCTTAAACCGGTTGAATCTTCAGATATGAAAATGACTCAGCTATTCACCAAAGTTGAATCAGAAGATACAAGTAGCCTCTTTGACAAACTTAAGAAGGAACCTGATGCTTTAACTTTGCTGGCCCCAGCCGCTGGAGACACAATCATATCTTTAGATTTTGGCAGCAACGACACAGAAACTGATGACCAGCAACTTGAGGAAGTACCATTATATAATGATGTAATGCTCCCCTCACCCAACGAAAAATTACAGAATATAAATTTGGCAATGTCTCCATTACCCACCGCTGAAACGCCAAAGCCACTTCGAAGTAGTGCTGACCCTGCACTCAATCAAGAAGTTGCATTAAAATTAGAACCAAATCCAGAGTCACTGGAACTTTCTTTTACCATGCCCCAGATTCAGGATCAGACACCTAGTCCTTCCGATGGAAGCACTAGACAAAGTTCACCTGAGCCTAATAGTCCCAGTGAATATTGTTTTTATGTGGATAGTGATATGGTCAATGAATTCAAGTTGGAATTGGTAGAAAAACTTTTTGCTGAAGACACAGAAGCAAAGAACCCATTTTCTACTCAGGACACAGATTTAGACTTGGAGATGTTAGCTCCCTATATCCCAATGGATGATGACTTCCAGTTACGTTCCTTCGATCAGTTGTCACCATTAGAAAGCAGTTCCGCAAGCCCTGAAAGCGCAAGTCCTCAAAGCACAGTTACAGTATTCCAGCAGACTCAAATACAAGAACCTACTGCTAATGCCACCACTACCACTGCCACCACTGATGAATTAAAAACAGTGACAAAAGACCGTATGGAAGACATTAAAATATTGATTGCATCTCCATCTCCTACCCACATACATAAAGAAACTACTAGTGCCACATCATCACCATATAGAGATACTCAAAGTCGGACAGCCTCACCAAACAGAGCAGGAAAAGGAGTCATAGAACAGACAGAAAAATCTCATCCAAGAAGCCCTAACGTGTTATCTGTCGCTTTGAGTCAAAGAACTACAGTTCCTGAGGAAGAACTAAATCCAAAGATACTAGCTTTGCAGAATGCTCAGAGAAAGCGAAAAATGGAACATGATGGTTCACTTTTTCAAGCAGTAGGAATTGGAACATTATTACAGCAGCCAGACGATCATGCAGCTACTACATCACTTTCTTGGAAACGTGTAAAAGGATGCAAATCTAGTGAACAGAATGGAATGGAGCAAAAGACAATTATTTTAATACCCTCTGATTTAGCATGTAGACTGCTGGGGCAATCAATGGATGAAAGTGGATTACCACAGCTGACCAGTTATGATTGTGAAGTTAATGCTCCTATACAAGGCAGCAGAAACCTACTGCAGGGTGAAGAATTACTCAGAGCTTTGGATCAAGTTAACTGA |
|  |  |
|  |  |
|  |  |
|  |  |
|  |  |
|  |  |
|  |  |
| sh-HIF-1α |  |
| sh1-HIF-1α | F:5’-ATCC**GCCGAGGAAGAA**CTATGAATTCAAGAGATTCATAGTTCTTCCTCGGCTTTTTTA－3’ |
|  | R:5’-GCTTAAAAAAGCCGAGGAAGAACTATGAATCTCTTGAATTCATAGTTCTTCCTCGGCG－3’ |
| sh2-HIF-1α | F:5’-GATCC**GCATTGTATGTGTGAATTA**TTCAAGAGATAATTCACACATACAATGCTTTTTTA－3’ |
|  | R:5’-AGCTTAAAAAAGCATTGTATGTGTGAATTATCTCTTGAATAATTCACACATACAATGCG－3’ |
| sh3-HIF-1α | F:5’-GATCC**GCTGGAGACACAATCATAT**TTCAAGAGAATATGATTGTGTCTCCAGCTTTTTTA－3’ |
|  | R:5’-AGCTTAAAAAAGCTGGAGACACAATCATATTCTCTTGAAATATGATTGTGTCTCCAGCG－3’ |
| sh-CON | F:5’-GATCC**GCCCAAGTATTAAATCAGA**TTCAAGACGTCTGATTTAATACTTGGGCTTTTTTA－3’ |
|  | R:5’-AGCTTAAAAAAGCCCAAGTATTAAATCAGACGTCTTGAATCTGATTTAATACTTGGGCG－3’ |

The underlined sequences represent the objective gene sequence
